# Psychological impact, support and information needs for women with an abnormal Pap smear: comparative results of a questionnaire in three European countries

**DOI:** 10.1186/1472-6874-11-18

**Published:** 2011-05-25

**Authors:** Joseph Monsonego, Javier Cortes, Daniel Pereira da Silva, Anna Francisca Jorge, Patrick Klein

**Affiliations:** 1 Institut of the Cervix, 174 rue de Courcelles, 75017 Paris, France; 2 Spanish Society of Obstetrics and Gynaecology, Alfonso el Magnánimo 29, 07004 Palma de Mallorca, Spain; 3 Cervical Pathology Unit, Portuguese Institute of Oncology in Coimbra, Santo António dos Olivais, 3030 Coimbra, Portugal; 4 Vision Critical, 61, rue de Monceau, 75008 Paris, France

## Abstract

**Background:**

Extensive information on cervical cancer is currently available. Its effectiveness in reducing anxiety in women receiving abnormal Pap tests is not clear. We investigated current practices of communicating abnormal Pap results to evaluate women's reactions and determine the sources of information they use subsequently.

**Methods:**

A self-administered questionnaire-based study was performed in 1475 women in France, Spain and Portugal who had received an abnormal Pap smear result in the 12 months prior to completing the questionnaire. Questions covered methods of communication of the result, emotional reactions, support received (from the physician and entourage), and information sources, using pre-specified check box options and rating scales. Data were analyzed by country.

**Results:**

Pap test results were mostly communicated by phone to Spanish women (76%), while physician letters were common in France (59%) and Portugal (36%). Frequent reactions were anxiety, panic and stress, which were less common in Spanish women than their French and Portuguese counterparts. After discussing with their physician, half of the participants were worried, despite rating highly the psychological support received. Over 90% of women in each country discussed their results with family or friends. Partners provided a high level of support. Overall, the abnormal diagnosis and consequences had a low to medium impact on daily, professional and family life and their relationships with their partner. Impact was higher in Spanish women than the French or Portuguese. Information on the diagnosis and its treatment was rated average, and nearly 80% of participants wanted more information, notably French women. Preferred sources were the physician and the Internet.

**Conclusions:**

Women expressed a strong wish for more information about cervical cancer and other HPV-related diseases, and that their physician play a major role in its provision and in support. There was a heavy reliance on the close entourage and the Internet for information, highlighting the need for dissemination of accurate material. Differences between countries suggest information management strategies may need to be tailored to different geographical regions.

## Background

Cervical cancer is the second most common cancer in women affecting nearly 530 000 females worldwide and resulting in 275 000 deaths each year, including 31 000 cases and 13 000 deaths in Europe [1]. Human papillomavirus (HPV) infection can lead to the development of pre-cancerous lesions and cervical cancer [2]. Routine screening and HPV vaccination programs have increased the visibility of cervical cancer in the wider community, and may contribute to increasing concerns about developing this cancer, particularly in younger women [3].

Studies report that after being informed of an abnormal Pap smear test result, women commonly feel stressed and anxious [4-6], irrespective of the severity of the result [7]. These emotions are often long lasting, being present up to two years after the Pap test [8]. Lack of accurate understandable medical information about the causes, prevention, treatment and consequences of an abnormal Pap smear result and cervical cancer leads to anxiety [9]. A study in the UK reported that the method and content of information communicated to patients receiving an abnormal smear varied widely, emphasizing the need for a uniform approach [10].

The introduction of national HPV vaccination programs and cancer screening campaigns in Europe has been highly publicized. Providing appropriate and accurate information is an important element in the successful management of cervical cancer screening. A study of over 1000 Irish women provided evidence of the negative impact of poor knowledge about Pap testing on screening attendance. False perceptions, including that the test is time consuming and that women having a test were at a higher risk of cervical cancer than those not having it, were barriers to women attending a routine screen [11]. Data on patient perception of abnormal Pap testing results subsequent to the implementation of these programs are limited, although a recent UK study described a need for better delivery and content of cervical screening result notifications and suggested that both screening centres and clinics need to review their practices [12].

Improving knowledge about cervical cancer in the wider community should contribute to reducing anxiety on receipt of an abnormal test. We performed a large questionnaire survey in Europe to assess "real life" experiences among women receiving an abnormal Pap smear test. We wished to identify patient's reactions to being informed, how well informed and supported they felt, and how they obtained information about their test result. Data were collected in relation to communication of results, women's reactions, support received, information about the potential consequences, as well as the impact this news had on their daily lives. This information can be used to improve communication and information practices.

## Methods

### Study design

We conducted a retrospective, cross-sectional, questionnaire-based study in which women were recruited from a total of 40 medical centres and gynaecological practices in France, Spain and Portugal between March and July 2008. The questionnaire was also distributed in Italy however data were incomplete and are not presented. The survey was implemented by the French Women Against Cervical Cancer (WACC) Foundation as part of a national education program on cervical cancer screening. Participating gynaecologists were selected by the WACC Foundation. The questionnaire was distributed to women consulting their physician for a routine visit or due to receipt of an abnormal test result following routine screening. Potential participants were given the questionnaire at the clinic reception on arrival for their consultation, and it was completed prior to their appointment. Women were free to refuse to complete the questionnaire. Ethics committee approval was not required for the study given that no clinical procedures were performed and patient records were not used.

### Participants

Potential participants were identified by the physician on the basis of a previous abnormal Pap result. Women consulting the gynaecologist for a routine visit or due to receipt of an abnormal test result following routine screening and who had received at least one abnormal Pap smear result (atypical squamous cells of undetermined significance [ASCUS] or more severe) during the previous 12 months were eligible to participate. Patients considered to have inadequate language skills or an intellectual disability were excluded. No other criteria were used.

### Questionnaire

The self-administered questionnaire was developed in English by a WACC Foundation task force on the basis of a literature review, and was translated into local languages. The questionnaire was validated by a country coordinator from each participating country in conjunction with an expert responsible for the methodology and analyses. Paper copies were completed with a pen in the waiting room prior to the consultation. An introductory paragraph explained to the participant why the WACC was performing the study and invited them to answer a series of questions (see Additional File 1: WACC Questionnaire). In brief, the questionnaire was composed of 15 questions covering basic demography and Pap test results, how participants were told about the abnormal Pap test result, how they felt when they were told, how they felt about the support they received from medical and personal networks, what treatment they had and how they obtained information about the result. Eleven of the questions had pre-specified check box response options and where appropriate, had a free-text option for 'other'. Responses to the question about diagnosis were cross-checked with the patient's medical dossier. Patients who did not know what diagnosis they had were not included. The number of answers allowed per question was specified. For the four questions regarding the psychological support received, impact on daily life, and how well informed they considered themselves to be, participants were asked to rank their response on a one-to-ten rating scale by circling the appropriate number; one was the least supported, informed or impacted while ten was completely supported, informed or impacted.

### Analysis

Data were entered in a central database and analyzed globally and by country. Descriptive statistics were used to describe all data. Results are presented by question using an abbreviated description. For questions with pre-specified response options, frequency by patient is presented according to pre-specified options. For questions with a rating scale, mean scores are presented with standard deviations. The corresponding survey question (see Additional File 1: WACC Questionnaire) is indicated in the text or table. The study was not designed for formal statistical analysis and data were thus reviewed for broad trends in current practices which may be used to identify areas for improvement. Student t-tests were performed to determine statistically significant differences between countries. Missing data were reported for between 2% and 18% of responses, depending on the question.

## Results

A total of 1475 women (765 in France, 467 in Spain and 243 in Portugal) with abnormal Pap smear results agreed to participate in the study. Demographic characteristics, summarised in Table 1, were similar between the three countries. The majority of participants (87%) were less than 50 years old. French and Spanish participants were younger compared to those from Portugal (35%, 29%, and 16% were younger than 30 years old, respectively). Just over half (55%) of the patients were married or living with their partner, and most (at least 61%) had at least one child. More French women were childless compared to women in Spain and Portugal (31%, 10% and 8%, respectively).

**Table 1:** Demographic characteristics of study participants

	% patients			
	**Total** **(N = 1475)**	**France** **(N = 765)** **A**	**Spain** **(N = 467)** **B**	**Portugal** **(N = 243)** **C**

**Age (years)**				
<30	26.6	34.9^C^*	28.6^C^	16.3
30 to 39	37.9	35.9	35.7	42.2
40 to 49	22.3	19.1	23.8	23.9
50 to 59	8.9	7.6	7.5	11.7
≥60	2.7	2.2	1.5	4.2
Missing data	1.6	0.3	2.9^A^	1.7
**Marital status**				
Married/living with partner	55.2	57.6	50	59.3
Single	32.3	33.3^C^	38.2^C^	24.1
Divorced	10.3	8.3	10.3	12.5
Widowed	2.2	0.8	1.5	4.1^A^
**Number of children**				
0	16.4	30.7^C^	10.4^C^	8.4
1	26.1	22.5	22.1	33.3
2	23.5	16.6	23.1	30.9^A^
>2	11.4	9.7	12.0	12.6
Missing data	22.6	20.5	32.4^AC^	14.8

Data from Question 1

Superscript letters (A, B, C) show significant superiority (0.1% risk; student t-test) relative to the corresponding value in the superscript column indicated. *eg, the 34.9% rate of patients under 30 years in France (column A) was significantly greater than the 16.3% rate in Portugal (column C).

### Communication of test results, follow-up, and feelings experienced

#### Method of communication of test results

Results of an abnormal Pap test were most commonly communicated to the participant via a telephone call (from the physician or the physician's secretary) to arrange an appointment to discuss the results (24% and 28% respectively, Table 2). Twenty-two percent of patients received a letter from the physician asking them to make an appointment. In some cases (14%), laboratory results were sent directly with a note from the physician. Results were different between countries; in Spain, approximately three-quarters of participants received the news over the phone, notably via the physician's secretary, while letters were rarely sent. In contrast, approximately 60% of French women and 40% of Portuguese patients were told via a letter.

**Table 2:** Communication of abnormal Pap test results, reaction to it (in at least 10% of patients in any one country) and follow-up of abnormal Pap smear results

	% patients			
	**Total** **(N = 1475)**	**France** **(N = 765)** **A**	**Spain** **(N = 467)** **B**	**Portugal** **(N = 243)** **C**

**Announcement made via**:				
**Letter**	**36.0**	**58.9**^**BC**^	**12.7**	**36.3**^**B**^
From physician	22.1	33.6^B^	9.2	23.5^B^
From laboratory ± physician	13.9	25.3^BC^	3.5	12.8^B^
**Phone call**	**52.0**	**31.9**	**75.7**^**AC**^	**48.4**^**A**^
From physician's secretary	27.8	8.5	53.4^AC^	21.5^A^
From physician	24.2	23.4	22.3	26.9
**Other**	**13.7**	**10.6**	**14.0**	**16.6**
Initial feelings after being told of abnormal Pap smear test result*				
Anxiety	59.1	74.0^B^	36.4	66.9^B^
Panic	32.9	29.1	25.6	43.9^AB^
Stress	23.3	38.6^BC^	7.6	23.9^B^
Did not know what it meant	22.9	25.1	29.9^C^	13.8
Incredulity	9.9	4.5	17.8^AC^	7.5
Guilty	8.6	8.4	11.9^C^	5.4
Anger	8.4	7.6^C^	15.6^AC^	2.0
Feelings after physician's explanation of findings and next steps*				
Worried	51.5	47.3	56.0^A^	51.2
Reassured	33.9	34.5	30.7	36.4
Confident	19.2	19.3^C^	7.3	31.0^AB^
Optimistic	17.7	9.8^BC^	19.9	23.5
Relieved	14.5	11.6	14.9	16.9
Disorientated	13.7	18.5^BC^	12.5	9.9
Depressed	11.8	15.7C	13.0^C^	6.6

Data from Questions 3, 4 and 5

Superscript letters (A, B, C) show significantly superiority (0.1% risk; Student t-test) relative to the corresponding value in the superscript column indicated.

*Up to three responses per question permitted

#### Feelings experienced upon being informed and support received

Overall, anxiety, panic and stress were the most frequent initial reactions (59%, 33% and 23% respectively; Table 2). Anger and guilt were reported in less than 10% of participants. Fewer Spanish women said they felt anxious (36%, versus 74% and 67% in France and Portugal respectively) or were stressed by the test results (8% compared to 39% of French women and 24% of Portuguese women). One in four women said they did not understand what the abnormal result meant and that they needed information, notably in France and Spain (25% and 30% respectively).

After having discussed the test results and follow-up treatment with their gynaecologist, approximately half of the patients remained worried, although a third said they felt reassured and nearly 20% felt confident (Table 2). Little variation was reported between countries in reactions to the physician's explanation of their results.

Patients felt that they had received reasonable psychological support from their gynaecologist in relation to being informed about the initial test results (Question 6), with a mean score of 7.3 (± 2.8) out of 10. Differences between countries in mean scores were apparent; 8.1 (± 2.2) in Portugal, 7.6 (± 2.8) in Spain and 6.2 (± 2.8) in France. In all three countries, the majority of women (93%) discussed their results with someone else (Question 7), notably their partner (69%), a friend (40%) or their mother (37%). The mean score for level of support received from a partner (Question 11) was 7.3 (± 3.1) out of 10, and was similar across countries, being 8.0 (± 2.7) in Portugal, 7.8 (± 2.9) in Spain and 6.8 (± 3.2) in France.

#### Impact on quality of life

Participants were asked to evaluate the impact of their illness (abnormal diagnosis and its consequences) on various aspects of their lives (Table 3). Overall, women rated the impact as being relatively low in all areas investigated; mean scores were 4.0 (± 3.0) in terms of their relationship with their partner, 3.4 (± 2.7) for daily life, 3.0 (± 2.5) for family life and 2.6 (± 2.4) for professional life. Portuguese women considered the diagnosis had a greater impact on their family and professional life compared to Spanish women (for family life, mean scores were 3.8 in Portugal compared to 2.6 in France and Spain; for professional life, mean scores were 3.1 compared to 2.5 and 2.4 in France and Spain, respectively).

**Table 3:** Impact of illness on aspects of life

	**Mean score (**±SD)*			
	**Total** **(N = 1475)**	**France** **(N = 765)** **A**	**Spain** **(N = 467)** **B**	**Portugal** **(N = 243)** **C**

**Has the illness had any impact on...?**				
Family life	3.0 (2.5)	2.6 (2.4)	2.6 (2.5)	3.8 (2.7)^AB^
Professional life	2.6 (2.4)	2.5 (2.3)	2.4 (2.5)	3.1 (2.5)
Daily life	3.4 (2.7)	3.4 (2.7)	3.1 (2.7)	3.6 (2.6)
Relationship with partner	4.0 (3.0)	4.0 (2.9)	3.9 (3.1)	4.1 (2.9)

Data from Question 10

Superscript letters (A, B, C) show significantly superiority (0.1% risk; student t-test) relative to the corresponding value in the superscript column indicated.

*Scale from 1 to 10, where 1 is no effect and 10 is severely affected.

### Information about the diagnosis and treatments

#### Diagnosis and treatment management

Diagnoses were mainly CIN1, 2/3, were not considered serious or were not in need of monitoring (approximately 25% each; Table 4). CIN1 or 2/3 diagnoses were at least twice as common in the Spanish and Portuguese participants than in the French women (70%, 59% versus 33% respectively), the latter having a higher rate of non-serious diagnoses (35%). The most common follow-up procedures prescribed were colposcopy, biopsy, 6-month cervical smear check-up or cervical conisation (50%, 42%, 32% and 30% respectively). Colposcopy was more common in France than Spain or Portugal, while check-up smears were prescribed more frequently in Spain. Approximately half of the participants were worried about the treatment plan proposed, compared to one-third who were reassured. Feelings evoked were similar across all three countries.

**Table 4:** Test results, participants' feelings towards treatment and impact on life

	% patients			
	**Total** **(N = 1475)**	**France** **(N = 765)** **A**	**Spain** **(N = 467)** **B**	**Portugal** **(N = 243)** **C**

**Abnormal test result**				
CIN1	28.4	16.6	39.7^A^	28.9^A^
CIN2/3	25.4	16.2	29.8^A^	30.2^A^
Not serious/to be monitored	24.8	34.6^BC^	16.2	23.7
Cervical cancer	4.7	2.6	3.3	8.2^A^
Other	14.4	22.8^BC^	13.2	7.3
Disease management*				
Colposcopy	49.5	70.7^BC^	35.6	42.1
Biopsy	42.0	32.1	44.4^A^	49.6^A^
Check-up smear in 6 months	31.6	26.2	44.2^AC^	24.4
Cervical conisation	30.0	20.0	38.0^A^	31.8^A^
HPV test	22.7	22.4	29.5^AC^	16.1
Check-up smear in 1 year	9.7	8.9	11.7	8.7
Other	9.3	8.9	8.6	10.3
Hysterectomy	2.1	0.8	2.6^A^	2.9
Don't know	3.1	3.4	2.2	3.7
Feelings about treatment or wait**				
Worried	55.9	50.0	53.9	63.9^A^
Confident (reassured)	33.8	31.3	34.2	35.7
Physical integrity under threat	15.7	21.8^BC^	14.7	10.5
Depressed	10.9	10.4	10.5	11.8
Disorientated	12.0	16.7^C^	13.4^C^	5.9
Ashamed	2.1	3.2	2.2	0.8
Alone	5.5	10.9^BC^	4.4	1.3

Data from Questions 2, 8, 9

Superscript letters (A, B, C) show significantly superiority (0.1% risk) relative to the corresponding value in the indicated superscript column.

*More than one response possible

**Up to three responses per question

#### Feelings about communication of information

Overall, in terms of being informed about their disease and treatments, mean scores were mid-range, being 5.9 (± 3.2) and 5.9 (± 3.3) respectively (Table 5). Scores were lower in France (4.8 ± 3.0 and 4.6 ± 3.0, respectively), compared with Spain (6.2 ± 3.4 and 6.1 ± 3.4, respectively) and Portugal (7.0 ± 2.8 and 7.0 ± 2.0, respectively). Women were also asked how well informed they felt about the consequences (emotional, on their relationship with their partner, family life and future motherhood) of their illness. Mean scores were also mid-range, being 4.8, 5.0, 4.9 and 5.2 respectively. French women consistently reported feeling less well-informed than Spanish or Portuguese women in all areas.

**Table 5:** Information on treatment and consequences of the disease

	Total (N = 1475)	France (N = 765) A	Spain (N = 467) B	Portugal (N = 243) C
Patients felt well-informed about the...*	**Mean score (**±SD)*			
Disease	5.9 (3.2)	4.8 (3.0)	6.2 (3.4)^A^	7.0 (2.8)^A^
Treatments	5.9 (3.3)	4.6 (3.0)	6.1 (3.4)^A^	7.0 (2.7)^AB^
Emotional consequences	4.8 (3.2)	3.1 (2.7)	5.1 (3.5)^A^	6.5 (2.8)^AB^
Consequences on your relationship	5.0 (3.3)	3.4 (2.8)	5.4 (3.5)^A^	6.3 (3.0)^AB^
Consequences on your family life	4.9 (3.3)	3.1 (2.8)	5.2 (3.5)^A^	6.5 (3.0)^AB^
Consequences on your future as a mother (fertility)	5.2 (3.5)	3.6 (3.1)	5.4 (3.7)^A^	6.6 (3.1)^AB^
Information source used **	**% patients**			
Doctor	79.6	67.4	85.4^A^	85.9^A^
Online	38.1	45.9^B^	33.8	34.7
Media	14.6	23.1^BC^	11.8	8.9
Friends	14.0	21.1^BC^	10.5	10.3
Family	10.5	13.9^BC^	7.2	10.3
Other	6.5	8.3	4.8	6.6
Potential best sources of information ***	**% patients**			
Doctor	79.0	77.0	72.7	87.4^AB^
Web site	40.9	49.7^B^	31.9	41.2
Newsletter	23.6	31.0^C^	29.4^C^	10.3
Media/TV	20.0	27.6^B^	7.6	24.8^B^
Women's group	9.7	9.8	11.3	7.9
Other	1.4	1.6	1.6	0.9

Data from Questions 12, 13, 15

Superscript letters (A, B, C) show significantly superiority (0.1% risk) relative to the corresponding value in the indicated superscript column.

* Scale from 1-10, where 1 is not at all informed and 10 is very well informed.

** Multiple responses possible.

*** Two responses possible.

#### Source of information

All women considered their gynaecologist to be the main source of information on the disease and its consequences (80%; Table 5). Internet was also important, being used by 38% of participants, notably in France (46%, versus 35% in Portugal and 34% in Spain), along with the media (television, radio, newspapers). Fewer French women obtained information from their physician than Spanish and Portuguese women. Friends and the media had a similar but low ranking as information sources (14% and 15% respectively). Participants had a preference for their physician and the Internet as information sources (79% and 41%, respectively), as well as newsletters and media (24% and 20% respectively; Table 5). When asked if they would like to receive more information on cervical cancer and other HPV-linked genital diseases and their prevention (Question 14), almost 80% of the women (76% France, 82% Spain and 82% Portugal) interviewed said they "absolutely" wanted more information.

## Discussion

Despite increasing public awareness of HPV and cervical cancer, the results from our survey of nearly 1500 women in France, Spain and Portugal show that the current profile of feelings (predominantly anxiety, panic and stress) in response to being informed of an abnormal Pap result corresponds with earlier published results [4-6]. Initial reactions of anxiety may be fueled by how this news is communicated. In our survey, test results were mainly communicated over the phone in Spain, while in Portugal and particularly in France, letters were more common. It has been reported that women receiving a diagnosis of CIN1, 2 or 3 over the phone wanted more information and the opportunity to discuss their results in a more personal way [13]. A recent report from Sweden suggested that the wording in a letter plays an important role in the level of anxiety felt by women referred for a colposcopy after an abnormal cervical smear [14].

Being well informed is critical for lowering anxiety over abnormal results. Concern in HPV-positive women waiting for a second HPV test was alleviated if their information needs were met [15]. In our study, approximately a quarter of the women did not understand what the test results meant and nearly 80% stated a wish for more information. The level of information provided was ranked as average, notably in terms of the consequences on their lives, emotions and family. Over half of the participants in our study remained worried following a discussion with their physician about what their test results meant. Furthermore, results from a study by Pirotta et al. [16] suggest that even when patients are well-informed they are not necessarily less anxious. This reinforces the need for continual reassurance and follow-up from their physician. While the majority of participants in our study reported favourably on the psychological support they received from their physician, an Australian study has shown that women wish to participate in decisions about their care but find it hard to ask questions [17]. Physicians should bear in mind that patients may not spontaneously request further information, despite (or perhaps because of) high levels of anxiety [9].

Our study highlights that in the current environment of easy access to information via the Internet, physicians are nonetheless considered the primary information provider. Furthermore, participants in all three countries expressed a preference for this. The fact that a high percentage of women said they also discussed their test results with their partners, friends or mothers should be taken into consideration. This emphasizes the importance of the general public being well informed about cervical cancer and precancerous diagnoses.

The method and content of the information communicated should be carefully considered. One study showed that women had a significantly higher level of satisfaction with visual teaching aids (graphic and video) compared with verbal communication [18]. Information sheets and verbal presentations about treatment, including details of procedures and long-term effects are helpful for reducing anxiety [19] and appear to be improving general knowledge [20]. In our study, over 40% of the women wished to use the Internet as an information source, however research into it is lacking. Physicians need to provide guidance on reliable Internet sources, and encourage patients to discuss the information they obtain. Care should also be taken to ensure that patients who do not use the Internet have adequate access to alternative information sources.

This study describes trends showing a need for improved information about abnormal Pap smear results in the wider European community. Nonetheless, it was not powered for statistical comparisons and the study design did not allow for statistical adjustment of results. As a consequence, factors such as participant age and marital status which differed between countries may affect the interpretation of results. Further investigations are needed to determine the impact of methods of communication, patient age and background on women's feelings, as well as what are the most effective sources of information, and how these factors differ between countries. Development of questionnaires such as the Process Outcome Specific Measure (POSM) questionnaire used in the TOMBOLA study will be important in further research [21].

Few studies on the psychological consequences of communication of abnormal Pap smear diagnoses in different countries are available. In this study, analysis of the results by country showed variations in perceptions and management of cervical cancer. Compared to women in Spain and Portugal, French women considered themselves to be less well-informed, less well-supported, were more anxious, and less satisfied with the level of information provided and the explanation of the potential consequences of the illness. Internet and media were used much more commonly in France than in other countries. This argues against a blanket communication strategy when informing patients of an abnormal Pap smear result, suggesting instead that information and management should be adapted on a country-specific basis.

## Conclusion

The study results confirm that psychological support when communicating an abnormal Pap smear result needs to be better managed to reduce women's anxiety. The predominant feeling of worry reported on receipt of an abnormal result and during follow-up, along with a clearly expressed wish for information on cervical cancer screening, show that there is room for improvement. Dissemination of accurate information to the general public is needed. Internet was ranked as an important information source, highlighting the need to guarantee the quality of this tool. Country differences suggest that policies should be tailored to different cultures.

## Competing interests

JM received funding to conduct studies in cervical cancer screening from Gen-Probe Inc. and in HPV vaccines from Merck and GlaxoSmithKline, and has participated in Steering Committees at Merck, and the Advisory Board of Sanofi Pasteur MSD, Gen-Probe, Qiagen, and Roche Diagnostics.

## Authors' contributions

JM conceived the study, participated in the design and drafted the manuscript. JC, DPS and AJ participated in the questionnaire design and recruited patients. PK participated in the questionnaire design and performed the statistical analysis. All authors read and approved the final manuscript.

## Pre-publication history

The pre-publication history for this paper can be accessed here:

http://www.biomedcentral.com/1472-6874/11/18/prepub

## Supplementary Material

Additional file 1 **WACC Questionnaire**. Copy of the WACC Questionnaire distributed to participants Click here for file
