# Supplementary material for: Psychological impact, support and information needs for women with an abnormal Pap smear: comparative results of a questionnaire in three European countries
Source: BMC Womens Health. 2011 May 25;11:18. doi: 10.1186/1472-6874-11-18 (PMC3123641; doi:10.1186/1472-6874-11-18)
Supplement: Additional file 1 — WACC Questionnaire. Copy of the WACC Questionnaire distributed to participants [file 1472-6874-11-18-S1.PDF]

Partner Logo

EUROGIN

VOICE of WOMEN

QUESTIONNAIRE

DO NOT DISCLOSE

WACC
WOMEN AGAINST CERVICAL CANCER

www.eurogin.com/2008/wacc

WOMEN AGAINST CERVICAL CANCER

Dear friend

Cervical cancer is the second leading cause of cancer mortality among women worldwide, and is caused by human papillomavirus infections. Yet there are a number of effective preventive strategies available today to protect women and to prevent disease progression, but they remain unfamiliar to most women.

At the Women Against Cervical Cancer Forum we strive to inform and educate health professionals, women and the public at large, about cervical cancer disease and its causes.

In addition to physical suffering, cervical diseases cause an immeasurable emotional and social despair among many affected women. Recognizing the impact of the disease on the lives of women and their families; recognizing the lack of proper information accessible to women; and recognizing the stigmatization of the reproductive tract diseases, we would like to take action today to improve access to medical information about cervical cancer prevention to the public at large.

To achieve that, we would like to assess the level of your knowledge and experience related to cervical diseases in general, and your views would be highly appreciated.

You will find enclosed here a series of 15 questions easy to answer and that will help us to be able to provide relevant information on women's health issues and cervical cancer prevention programs, to improve participation of women in prevention programs in the near future.

We count on you and greatly appreciate your participation.

Warm regards,

J.Monsonego
EUROGIN Chairman

How to get more information: www.eurogin.com/2008/wacc

1. Your background:

Age: |__|__| Country:..

Marital status:

- ❑ Married, living with partner
- ❑ Single
- ❑ Divorced
- ❑ Widowed

Number of children |__|__|

2. Please state the diagnosis given by your doctor:

Only one answer possible

- ❑ Nothing serious but requires monitoring
- ❑ Cervical lesion (CIN1)
- ❑ Cervical lesion (CIN2 / CIN3)
- ❑ Cervical cancer
- ❑ Other...
- ❑ Don't remember

3. How did you find out about the results of your "abnormal" smear test?

Only one answer possible

- ❑ You were sent a letter from your doctor asking you to make an appointment to see him regarding the results of your smear test
- ❑ You were sent a letter with the results from the laboratory and an additional note from your doctor
- ❑ Your doctor called to inform you of the results of your smear test
- ❑ The doctor's secretary called to arrange an appointment for you to discuss the results of your smear test with your doctor
- ❑ Other...

WACC
WOMEN AGAINST CERVICAL CANCER

4. When you received the results of your abnormal smear, what was the first thing you felt?

Please, select no more than 3 answers

- ❑ Anxiety
- ❑ Panic
- ❑ Anger
- ❑ Alone
- ❑ Stress
- ❑ Incredulity
- ❑ Guilty
- ❑ Injustice
- ❑ Shame
- ❑ Didn't know what it meant (need for further information)
- ❑ Nothing special
- ❑ Other..

5. After your doctor had explained what the diagnosis meant and what the next steps will be, how did you feel? Did you feel…

Please, select no more than 3 answers

- ❑ Reassured
- ❑ Worried
- ❑ Optimistic
- ❑ Aggressive
- ❑ Confident
- ❑ Depressed
- ❑ Relieved
- ❑ Helpless
- ❑ Disorientated
- ❑ Ashamed
- ❑ Other..

WACC
WOMEN AGAINST CERVICAL CANCER

6. On a scale of 1 to 10 with regard to your relationship with your doctor how would you rate the psychological support that you received from your doctor throughout this examination? (where 1 means that you felt extremely alone and 10 that you felt completely supported by your doctor)

Please, circle the most appropriate number for you

Extremely alone 1 2 3 4 5 6 7 8 9 10 Completely supported

7. After you were given the results by your doctor and / or your treatment, did you talk about your disease with somebody else?

- ❑ **No**
- ❑ **Yes (precise):**
  - ❑ my partner
  - ❑ my friend
  - ❑ my mother
  - ❑ another member of my family
  - ❑ my daughter
  - ❑ other...

8. What treatment did you receive?

Multiple answers possible

- ❑ **Check-up smears for 6 months**
- ❑ **Check-up smears for 1 year**
- ❑ **HPV test**
- ❑ **Colposcopy**
- ❑ **Biopsy**
- ❑ **Cervical conisation**
- ❑ **Hysterectomy**
- ❑ **Don't know**
- ❑ **Other ....**

WACC

WOMEN AGAINST CERVICAL CANCER

9. How did you manage the treatment or wait?

Please, select no more than 3 answers

- ❑ I was very worried
- ❑ I was confident (reassured)
- ❑ I was depressed
- ❑ I felt that my physical integrity was under threat
- ❑ I was disoriented
- ❑ I was ashamed
- ❑ I felt alone

10. Has your illness had any impact on:

(where 1 means that you felt not at all affected and 10 that you felt severely affected)

On each line please circle the most appropriate number for you

	not at all affected								severely affected
Your family life?	1	2	3	4	5	6	7	8	9	10
Your relationship with your partner?	1	2	3	4	5	6	7	8	9	10
Your daily life?	1	2	3	4	5	6	7	8	9	10
Your professional life?	1	2	3	4	5	6	7	8	9	10

11. On a scale of 1 to 10 with regard to your relationship with your partner how would you rate the psychological support that you received from your partner throughout this examination?

(where 1 means that you felt extremely alone and 10 that you felt completely supported by your partner)

Please, circle the most appropriate number for you

Extremely alone 1 2 3 4 5 6 7 8 9 10 Completely supported

12. More precisely, on a scale from 1 to 10, would you say that you were well informed on

(where 1 means not informed at all and 10 means very well informed)

On each line please circle the most appropriate number for you

	not at all informed									very well informed
The illness	1	2	3	4	5	6	7	8	9	10
The treatments	1	2	3	4	5	6	7	8	9	10
The emotional consequences	1	2	3	4	5	6	7	8	9	10
The consequences on your relationship	1	2	3	4	5	6	7	8	9	10
The consequences on your family life	1	2	3	4	5	6	7	8	9	10
The consequences on your future as a mother (fertility)	1	2	3	4	5	6	7	8	9	10

13. Where did you get the information from?

Multiple answers possible

- ❑ Media
- ❑ Friends
- ❑ Family
- ❑ Doctor
- ❑ On line
- ❑ Other

14. Would you like more information on cervical cancer and other genital diseases linked to papillomavirus and on the way to prevent them?

Only one answer possible

- ❑ Yes, absolutely
- ❑ Yes, maybe
- ❑ Not really
- ❑ Definitely not

15. What would be the most useful sources of information for you?

Please select 2 answers

- ❑ Website
- ❑ Newsletter
- ❑ My doctor
- ❑ Women's groups
- ❑ Media / TV
- ❑ Other

WACC

WOMEN AGAINST CERVICAL CANCER

WACC

WOMEN AGAINST CERVICAL CANCER

www.eurogin.com/2008/wacc
